# Biochemical Characterization of Prion Strains in Bank Voles 

**DOI:** 10.3390/pathogens2030446

**Published:** 2013-07-02

**Authors:** Laura Pirisinu, Stefano Marcon, Michele Angelo Di Bari, Claudia D’Agostino, Umberto Agrimi, Romolo Nonno

**Affiliations:** Department of Veterinary Public Health and Food Safety, Istituto Superiore di Sanità, Viale Regina Elena, Rome 299-00164, Italy; E-Mails: stefano.marcon@iss.it (S.M.); michele.dibari@iss.it (M.A.D.B.); claudia.dagostino@iss.it (C.D.A.); umberto.agrimi@iss.it (U.A.); romolo.nonno@iss.it (R.N.)

**Keywords:** prion, prion strain, TSE, PrP^Sc^, bank vole, CJD, scrapie, BSE, conformational stability, GdnHCl

## Abstract

Prions exist as different strains exhibiting distinct disease phenotypes. Currently, the identification of prion strains is still based on biological strain typing in rodents. However, it has been shown that prion strains may be associated with distinct PrP^Sc^ biochemical types. Taking advantage of the availability of several prion strains adapted to a novel rodent model, the bank vole, we investigated if any prion strain was actually associated with distinctive PrP^Sc^ biochemical characteristics and if it was possible to univocally identify strains through PrP^Sc^ biochemical phenotypes. We selected six different vole-adapted strains (three human-derived and three animal-derived) and analyzed PrP^Sc^ from individual voles by epitope mapping of protease resistant core of PrP^Sc^ (PrP^res^) and by conformational stability and solubility assay. Overall, we discriminated five out of six prion strains, while two different scrapie strains showed identical PrP^Sc^ types. Our results suggest that the biochemical strain typing approach here proposed was highly discriminative, although by itself it did not allow us to identify all prion strains analyzed.

## 1. Introduction

Transmissible spongiform encephalopathies (TSEs), or prion diseases, are neurodegenerative disorders that afflict humans and others mammals. Prion diseases may be caused by prion exposure (acquired forms), mutations in PRNP gene (genetic or hereditary forms) and sporadic events in which the source of infection has not yet been demonstrated (idiopathic or sporadic forms). They include sporadic and genetic Creutzfeldt-Jakob disease (sCJD and gCJD) in humans, scrapie in sheep and goats and bovine spongiform encephalopathy (BSE) in cattle.

All TSEs are characterized by the accumulation of PrP^Sc^, the pathological form of host encoded prion protein (PrP^C^), considered to be the main or sole component of infectious agent termed prion, according to the protein-only hypothesis [1]. 

Prions exist as different strains that, when propagated in the same host, exhibit distinct disease phenotypes that persist upon serial transmissions [2,3]. Within the context of the protein-only hypothesis, it has been suggested that prion strain diversity is encrypted in distinct conformations of PrP^Sc^ aggregates [4]. 

Although the identification of prion strains is still based on biological strain typing in rodents, several studies showed that prion strains can be distinguished based on different biochemical properties of PrP^Sc^: the electrophoretic features of the protease resistant core of PrP^Sc^ [5,6,7], the relative proteinase K resistance of PrP^Sc^ [8,9], or the physico-chemical behavior of PrP^Sc^ during denaturation [10,11,12]. Indeed, such biochemical discrimination allowed large scale testing of small ruminants TSEs in EU in order to recognize the possible presence of BSE [13,14,15,16,17]. Notwithstanding, no unequivocal relationship between type of PrP^Sc^ and strain has been demonstrated so far, possibly due to technical difficulties which do not allow the direct structural analysis of PrP^Sc^ aggregates, or due to the presence of still unidentified strain-specific co-factors [18].

Further drawbacks may derive by PrP^res^-based approaches, as these are focused on the PK-resistant core of PrP^Sc^ (PrP^res^), while it is becoming increasingly clear that protease-sensitive isoforms of PrP are involved in different animal and human prion diseases [12,19,20,21,22,23]. These findings led us to develop a new method (CSSA) which does not rely on the protease resistant PrP^Sc^ but allows us to study the conformational properties of both protease sensitive and protease-resistant PrP^Sc^ species [11]. 

We have shown that bank voles (Myodes glareolus) are susceptible to a wide range of prion sources [24,25,26,27,28,29]. Taking advantage of vole-adapted prion strains, this study aimed at investigating if any different strain is actually associated with distinctive PrP^Sc^ types and if it might be possible to unequivocally identify strain through these biochemical phenotypes. We selected vole-adapted human and animal strains including gCJD and sCJD subtypes, sheep classical scrapie and bovine BSE. PrP^Sc^ from voles was analyzed by semi-quantitative western blot after PK digestion (PrP^res^) and after denaturation with increasing GdnHCl concentrations and PrP^C^/PrP^Sc^ differential centrifugation (conformational stability and solubility assay).

## 2. Results and Discussion

### 2.1. Selection of Vole-Adapted Strains

As previously reported, the bank voles are susceptible to a wide range of prion sources: human sCJD and gCJD [24,25], sheep scrapie [26,27], cattle and sheep BSE [24,28], cervid Chronic Wasting Disease [29] and mouse- and hamster-adapted scrapie strains [24,30]. With the aim of studying the strain-specific biochemical features of PrP^Sc^, we selected 43 brain tissues from 10 vole-adapted isolates representative of six different vole-adapted prions strains, three human-derived and three animal-derived: (i) sCJD MM1, MV1 and gCJD E200K, all belonging to the same vole-adapted strain “CJD type 1” [24]; (ii) sCJD MM2 [24]; (iii) sCJD MV2; (iv) Italian sheep scrapie SS7 [26]; (v) UK sheep scrapie isolates SCR1, SCR10 and SCR11 [26,31] which all gave in vole the same scrapie strain, “UK85”, different from that derived from Italian scrapie isolates, It93; (vi) bovine BSE [28]. With the exception of sCJD MV2, SCR1, SCR10 and SCR11, which will be reported elsewhere (manuscript in preparation), the transmission features of all these isolates (attack rates, survival times and lesion profiles), have been previously reported [24,26,28].

### 2.2. PrP^res^-Epitope Mapping

**Figure 1:**
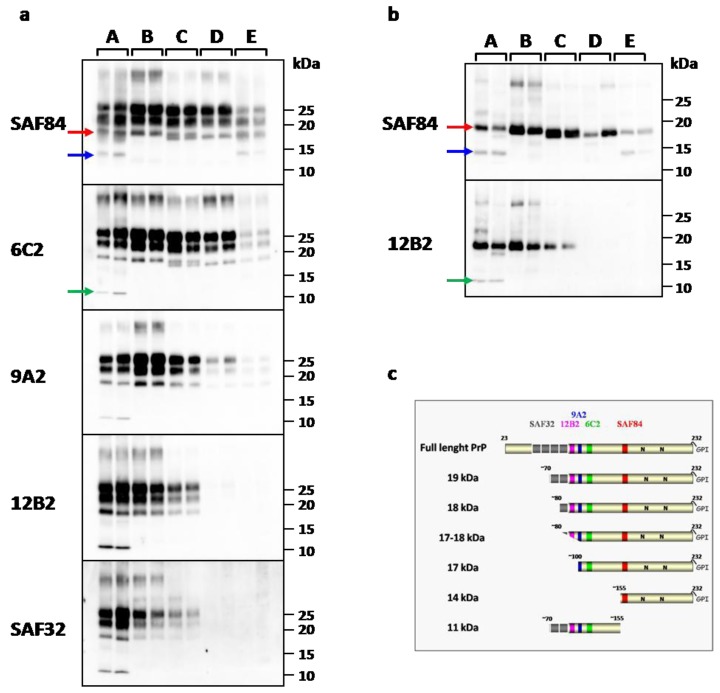
**(a)** Western Blot analysis of PK resistant PrP^Sc^ in representative vole-adapted prion strains. Replica blots were probed with different monoclonal antibodies indicated on the left of each blot. Representative samples for each PrP^res^ type were loaded from the highest (type A) to the lowest (types D and E) MW, as reflected by the progressive loss of *N*-terminal epitopes. PrP^res^ types are indicated on the top of the blots. Type A (lanes 1-2): CJD type 1; type B (lanes 3-4): sheep scrapie It93 and UK85; type C (lanes 5-6): sCJD MV2; type D (lanes 7-8): BSE; type E (lanes 9-10): sCJD MM2; **(b)** Samples shown in (a) after deglycosylation. (a,b) Red Arrow: 19 kDa fragment; blue arrow: 14 kDa fragment; green arrow: 11 kDa fragment. (c) Schematic representation of full length PrP and PrP^res^ fragments identified by epitope mapping and deglycosylation. The cleavage sites were determinate by the presence or absence of epitopes examinated (a). The presence of glycosylation sites were confirmed by deglycosylation treatment (b). The location of SAF32, 12B2, 9A2, 6C2, SAF84 mAbs used for the PrP^res^-epitope mapping are shown.

PrP^res^-epitope mapping was performed on protease treated PrP^Sc^ analyzed by western blot with a large panel of mAbs spanning the bank vole PrP, before and after deglycosylation (Figure 1a,b). This sensitive technique allowed the identification of several PrP^res^ fragments which were identified based on the presence or absence of the epitopes examined (Figure 1c). Overall, we identified five different patterns of PrP^res^, each characterized by the presence of one or more specific PrP^res^ fragments, that were labeled from A to E (Table 1). 

**Table 1:** Classification of prion strains in bank voles based on PrP^res^ analysis.

Type of PrP^res^	Strain	Inoculum ^1^
**A** (19 kDa + 14 kDa + 11 kDa)	CJD type 1	sCJD MM1
		sCJD MV1
		gCJD E200K
**B** (18 kDa)	Scrapie It93	SS7
**B** (18 kDa)	Scrapie UK85	SCR1
		SCR10
		SCR11
**C** (17–18 kDa)	CJD MV2	sCJD MV2
**D** (17 kDa)	BSE	BSE
**E** (17 kDa + 14 kDa)	CJD MM2	sCJD MM2

^1^ MM, MV, or VV indicate genotype at codon 129; 1 or 2 indicate the molecular types, classified according to Parchi *et al*. [6]

The type A was found only in voles infected with sCJD MM1/MV1 and gCJD E200K, and was characterized by three PrP^res^ fragments of 19, 14 and 11 kDa (Figure 1a,b). The main 19 kDa fragment was detectable equally well by all mAbs, while the 14 kDa fragment was recognized only by SAF84 (Figure 1a,b). Thus 19 kDa and 14 kDa were both *C*-terminal fragments with a different PK cleavage site at their *N*-terminus and included the glycosylation sites with the corresponding mono-glycosylated and di-glycosylated bands, resulting in a complex electrophoretic pattern when the membrane was probed with SAF84. In contrast, the 11 kDa fragment was recognized by 6C2, 9A2, 12B2 and SAF32 but not by SAF84, thus indicating that it is an internal fragment cleaved at both the *N* and *C*-termini (Figure 1a,b). 

The PrP^res^ type B, was found in brain homogenates from bank voles infected with classical scrapie isolates. It was characterized by a single *C*-terminal fragment of 18 kDa (unglycosylated band): this fragment was recognized by all mAbs, but only partially by SAF32 (Figure 1) indicating that the cleavage site was more *C*-terminal respect to the type A fragment of 19 kDa.

The type C, characteristic of sCJD MV2 PrP^res^, showed a molecular weight intermediate between type B and D, which was well detectable with SAF84 and 6C2. With these two antibodies, sometimes the PrP^res^ appeared as a 17–18 kDa doublet (Figure 1a). Type C PrP^res^ was still recognized by mAbs *N*-terminal to 6C2, although with lower affinity and without showing the PrP^res^ doublet (Figure 1a).

The PrP^res^ type D, found in BSE, was characterized by a single *C*-terminal fragment of 17 kDa which was well detectable with SAF84 and 6C2, but barely detected by 9A2, and not at all by 12B2 and SAF32 mAbs (Figure 1a,b).

The type E, found only in voles inoculated with sCJD MM2, showed a PrP^res^ characterized by two PK-resistant fragments of 17 and 14 kDa. The main PrP^res^ fragment was a 17 kDa similar to type D, being well recognized only by SAF84 and 6C2. However, compared to type D, it was clearly less glycosylated (Figure 1a). The 14 kDa fragment was *C*-terminal and glycosylated too, being similar to the 14 kDa PrP^res^ fragment described above in type A PrP^res^.

Overall, the analysis of PrP^res^ revealed a close correspondence with each strain, allowing us to discriminate all human-derived strains among them and from animal strains (scrapie and BSE). However, voles infected with two different scrapie strains showed identical PrP^res^ patterns. 

Interestingly, the PrP^res^ pattern of scrapie, BSE, and sCJD subtypes, seems well preserved after transmission to voles. As previously reported [24], voles infected with sCJD subtypes faithfully reproduced the PrP^res^ electrophoretic mobilities of human counterparts. Also in this study, with more detailed biochemical analyses, we confirmed that vole-adapted sCJD MM1/MV1 had a lower electrophoretic mobility than sCJD MM2 (19 kDa *vs*. 17 kDa) due to a different *N*-terminal PK cleavage site. Moreover, additional *C*-terminal fragments were found to be associated to sCJD subtypes [32,33] as here found in bank voles inoculated with sCJD MM1/MV1 and MM2. In addition, sCJD MV2 adapted to voles showed an intermediate molecular weight between sCJD MV1/MM1 and MM2 and displayed a unique doublet band similar to that described in human sCJD MV2 cases [34]. Furthermore, as observed in natural isolates, in voles the scrapie PrP^res^ was cleaved by PK more *N*-terminally than PrP^res^ from BSE adapted to vole (18 kDa *vs*. 17 kDa), as confirmed by differential detection of 12B2 and SAF32 mAbs.

### 2.3. PrP^Sc^ Conformational Stability

As previously reported [11], the conformational stability analysis of PrP^Sc^ from voles infected with sCJD and gCJD showed distinct susceptibilities to denaturation. Indeed, sCJD MM1/MV1 and gCJD E200K showed the highest resistance to guanidine with [GdnHCl]_1/2_ values > 2.8 M while sCJD MM2 was the most susceptible with [GdnHCl]_1/2_ value of 1.6 M and scrapie (SS7) displayed intermediate susceptibility (2.1 M) [11]. Here we extend these findings by analyzing a further sCJD derived strain, sCJD MV2, a second vole-adapted scrapie strain, called UK85, and BSE (Table 1). PrP^Sc^ from voles infected with sCJD MV2 showed intermediate susceptibility to denaturation respect to MV1/MM1/E200K and MM2 CJD, with [GdnHCl]_1/2_ value of 2.2 ± 0.1 M. PrP^Sc^ from voles infected with scrapie UK85 strain showed [GdnHCl]_1/2_ value of 2.0 ± 0.2 M comparable with that observed from scrapie SS7 strain. The [GdnHCl]_1/2_ value of BSE PrP^Sc^ was 2.4 ± 0.2 M. 

Overall, the conformational stability analysis allowed us to discriminate all sCJD subtypes based on their relative susceptibility to denaturation. Moreover, BSE showed a higher resistance to denaturation than scrapie confirming that observed in scrapie and BSE natural isolates [35]. Nevertheless, as observed by PrP^res^ analysis, conformational stability analysis did not enable us to distinguish the two scrapie strains. 

**Figure 2:**
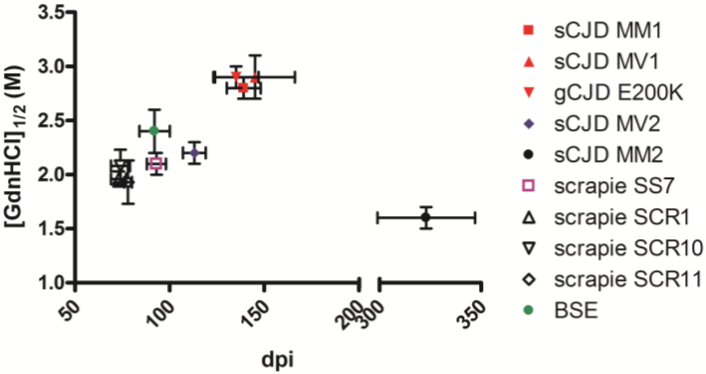
Graph of survival times (day post inoculation, dpi) and the conformational stability of PrP^Sc^ ([GdnHCl]_1/2_) of human and animal isolates transmitted to bank voles. Mean survival times ± SD after the third passage were: sCJD MM1, 139 ± 9; sCJD MV1, 145 ± 21; gCJD E200K, 135 ± 12; sCJD MV2, 113 ± 6 (second passage); sCJD MM2, 323 ± 24; scrapie SS7, 93 ± 5; scrapie SCR1, 73 ± 4; scrapie SCR10, 74 ± 5; scrapie SCR11, 78 ± 2 ; BSE, 92 ± 8 [28]. The [GdnHCl]_1/2_ values (mean ± SD) obtained by CSSA were: sCJD MM1, 2.8 ± 0.1 [11]; sCJD MV1, 2.9 ± 0.2 [11]; gCJD E200K, 2.9 ± 0.1 [11]; sCJD MV2, 2.2 ± 0.1; sCJD MM2, 1.6 ± 0.1 [11]; scrapie SS7, 2.0 ± 0.1 [11]; scrapie SCR1, 2.0 ± 0.1; scrapie SCR10, 2.1 ± 0.1; scrapie SCR11, 1.9 ± 0.2 ; BSE, 2.4 ± 0.2.

Previous studies suggested that the conformational stability of PrP^Sc^ aggregates correlated with the incubation time of prion strains, in particular, less stable mice prions replicated more rapidly [36]. On the contrary, a further study reported that short incubation period strains in hamster were characterized by more stable PrP^Sc^ [37]. We investigated the relationship between survival times and PrP^Sc^ conformational stability in bank vole strains and did not find a clear correlation (Figure 2). If these discrepancies depend on the different animal models used in these studies or on other factors, such as the strain-specific capacity for neuroinvasion [38] and/or neuropathological characteristics [37], will be further investigated. 

## 3. Experimental Section

### 3.1. Brain Tissues

We selected brain tissues from voles infected with six different vole-adapted prion strains deriving from transmissions of human and animal prions. All tissues analyzed were from vole expressing the PrP variant with met 109 [30]. Brain tissues were derived from second or third vole passages of sCJD MM1 (*n* = 4), sCJD MV1 (*n* = 3), sCJD MM2 (*n* = 5), sCJD MV2 (*n* = 5), gCJD E200K (*n* = 3), BSE (*n* = 5), Italian sheep scrapie SS7 (*n* = 4) and UK sheep scrapie cases SCR1 (*n* = 4), SCR10 (*n* = 4) and SCR11 (*n* = 6).

### 3.2. PrP^res^ Analysis

Brain homogenates (20% *w*/*v*) were prepared in 100 mM Tris-HCl with Complete protease inhibitor cocktail (Roche) at pH 7.4. The homogenates were either used directly or stored at −20 °C. After adding an equal volume of 100 mM Tris-HCl containing 4% sarkosyl, the homogenates were incubated for 30 min at 37 °C with gentle shaking. Proteinase K (Sigma-Aldrich) was added at a final concentration of 250 μg/mL and then the samples were incubated for 1 h at 55 °C with gentle shaking. The reaction was stopped with 3 mM PMSF (Sigma-Aldrich, St. Louis, MO, USA). Aliquots of samples were added with an equal volume of isopropanol/butanol (1:1 *v*/*v*) and centrifuged at 20,000 g for 5 min. The supernatant were discarded and the pellets were re-suspended in denaturing sample buffer (NuPage LDS Sample Buffer and NuPage Sample Reducing Agent, Invitrogen, Carlsbad, CA, USA) and were analyzed by Western Blotting.

Deglycosylation was performed by adding 18 μL of 0.2 M sodium phosphate buffer (pH 7.4) containing 0.8% Nonidet P40 (Roche) and 2 μL (80 U/mL) of N-Glycosidase F (Roche) to 5 μL of denaturated samples and by incubating overnight at 37 °C with gentle shaking. Samples were then analyzed by Western blotting.

### 3.3. Conformational Stability Analysis

As previously described [11], the conformational stability was analysed by CSSA. Briefly, aliquots of brain homogenates (6% *w*/*v* in 100 mM TrisHCl, pH 7.4) were added with an equal volume of 100 mM TrisHCl (pH 7.4), sarcosyl 4% and incubated for 1 h at 37 °C with gentle shaking. Then, aliquots of each sample were incubated for 1 h at 37 °C with different concentrations of GdnHCl (Pierce) to obtain final concentrations ranging from 0 to 4 M. After GdnHCl treatment samples were centrifuged at 20,000 g for 1 h at 22 °C and the pellets were re-suspended in denaturing sample buffer (NuPage LDS Sample Buffer and NuPage Sample Reducing Agent, Invitrogen) and analysed by WB. The dose-response curves allowed us to estimate the concentration of GdnHCl able to solubilize 50% of PrP^Sc^ (GdnHCl_1/2_). Individual denaturation curves were analyzed and best-fitted by plotting the fraction of PrP^Sc^ remaining in the pellet as a function of GdnHCl concentration, and using a four parameter logistic equation (GraphPad Prism). 

### 3.4. Western Blotting

Electrophoresis and Western blotting were performed as previously described [24]. Briefly, samples were denatured by adding NuPage LDS Sample Buffer (Invitrogen, Carlsbad, California, United States) and NuPage Sample Reducing Agent (Invitrogen), and heating for 10 min at 90 °C. After centrifugation at 10,000 g for 5 min each sample was loaded onto 12% bis-Tris polyacrylamide gels (Invitrogen). After electrophoresis and Western blotting on PVDF membranes (Immobilon-P; Millipore, Bedford, MA, USA), the blots were processed by SNAP i.d.TM Protein Detection System (Millipore, Bedford, MA, USA) as described by the manufacturer instructions. The monoclonal antibodies used, their epitopes on bank vole PrP and the working dilutions were as follow: SAF84, PrP residues 163–169, 1.2 μg/mL; 6C2, PrP residues 111–116, 8.6 μg/mL; 9A2, PrP residues 99–101, 1.2 μg/mL; 12B2, PrP residues 89–93, 2.4 μg/mL and SAF32, PrP octarepeat, 2.4 μg/mL. 

Following incubation with horseradish peroxidase-conjugated anti-mouse immunoglobulin (Pierce Biotechnology, Rockford, IL, USA) at 1:13,000, the PrP bands were detected by enhanced chemiluminescent substrate (SuperSignal Femto, Pierce, Rockford, IL, USA) and VersaDoc imaging system (Bio-Rad). The chemiluminescence signal was quantified by QuantityOne software (Bio-Rad).

## 4. Conclusions

This study confirms that by using appropriate biochemical methodologies it is possible to obtain indirect information on the strain-specific conformation of PrP^Sc^, such to allow a strain typing approach with a quite high discriminatory power. Indeed, five out of six prion strains analyzed were characterized by specific PrP^Sc^ types, clearly distinguishable by the combined analyses of PrP^res^ fragments and PrP^Sc^ conformational stability. As such, our data further support the close relationship between TSE strains and PrP^Sc^ properties [4]. However, even within our experimental setting in which tested samples were represented by known, rodent-adapted prion strains, it was not possible to discriminate all prion strains analyzed solely based on the biochemical characterization of PrP^Sc^. Indeed, all vole adapted scrapie isolates showed indistinguishable PrP^Sc^ types, although belonging to 2 different vole-adapted scrapie strains, endowed with different survival times (Figure 2) and neuro-pathological phenotypes (data not shown). If this simply reflects our inability to detect conformational differences by an indirect biochemical approaches, or has a broader implication in term of the relationships between PrP^Sc^ conformations and prion strains is currently under investigation. 

Recently we have applied the biochemical PrP^Sc^ typing methods here developed to the study of atypical human and small ruminant prions [39]. Interestingly, we confirmed a very high ability to discriminate PrP^Sc^ types, even in prion diseases known to be enriched in PK-sensitive PrP^Sc^ aggregates, such as VPSPr and Nor98 [23,39]. Indeed, it was possible not only to discriminate PrP^Sc^ types based on the prion disease, but also to associate specific PrP^Sc^ types to different PrP mutations in Gerstmann-Sträussler-Scheinker (GSS). 

Ongoing studies with other vole-adapted strains and alternative biochemical approaches will hopefully allow to further increase the discriminatory power of PrP^Sc^ typing. Although the lack of discrimination of the two scrapie strains, we believe that our study proposes a relatively easy and straightforward biochemical approach useful to refine the biochemical strain typing in animal models and in natural prion isolates.
